# Application of graphene oxide in the adsorption and extraction of bioactive compounds from lemon peel

**DOI:** 10.1002/fsn3.2363

**Published:** 2021-06-02

**Authors:** Valeh Sharif Nasirian, Seyed‐Ahmad Shahidi, Hasan Tahermansouri, Fereshteh Chekin

**Affiliations:** ^1^ Department of Food Science and Technology Ayatollah Amoli Branch Islamic Azad University Amol Iran; ^2^ Department of Chemistry Ayatollah Amoli Branch Islamic Azad University Amol Iran

**Keywords:** adsorption, flavonoids, graphene oxide, mechanism, rutin

## Abstract

The bioactive compounds like rutin, naringin, and gallic acid have been separated from lemon peel by graphene oxide (GO). The different influences such as pH values and separation conditions were investigated. Moreover, the samples were characterized by Fourier transform infrared spectroscopy, thermogravimetric analysis, scanning electron microscopy, UV‐Vis spectroscopy, and high‐performance liquid chromatography. The findings of high‐performance liquid chromatography revealed that the adsorbed proportion of rutin by GO was more than naringin and gallic acid so that 66.7% of rutin, 34% of naringin, and 19% of gallic acid from the extract were remarkably adsorbed and separated. Besides, adsorption percentage of these materials by GO was considered 74.8% after five cycles of adsorption–desorption process. On the other hand, we carried out batch experiments in order to study the adsorption mechanism of rutin on the GO since rutin was the highest quantity of bioactive substance in lemon peel. Pseudo‐second‐order kinetic model and Langmuir isotherm were the best models for describing adsorption process of rutin by GO. Adsorption capacity of rutin by GO was obtained about 21.08 mgg^−1^. In addition, the physical adsorption of rutin by GO was confirmed by Dubinin–Radushkevich isotherm. This research confirmed that this method for separation of flavonoids is simple and less cost.

## Graphical abstract

The simultaneous extraction of rutin, naringin, and gallic acid from lemon peel using GO was studied for the first time. The results showed that the adsorbed proportion of rutin by GO was more than naringin and gallic acid so that 66.7% of rutin, 34% of naringin, and 19% of gallic acid from the extract were remarkably separated.

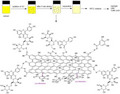

## 1 INTRODUCTION

Citrus fruits like lemon are widely consumed universal since they can help to improve the health of people (Benavente‐Garcia & Castillo, 2008). It was recognized that lemon not only prohibits the different diseases such as hypertension and tumor, but also can be even applied as a fuel or catalyst (Adibelli et al., 2009; Manjunatha et al., 2018; Musawwer Khan et al., 2018; Talib, 2017). This topic can be related to the presence of many bioactive groups in lemon. These compounds involve the significant proportion of the flavonoids, which are the phenolic natural substances, and other useful ingredients such as vitamins (Panche et al., 2016; Stefova et al., 2003). Lately, an article was published about the activity of these natural products on the COVID‐19 (Silva Antonio et al., 2020). On the other hand, rutin and naringin are the plentiful flavonoids in citrus fruits (Wang et al., 2008). These flavonoids were generally applied as antioxidant (El‐desoky et al., 2018; Mallepu et al., 2018). Moreover, a huge number of reports have been presented about the medical activities of naringin and rutin (El‐desoky et al., 2018; Gullón et al., 2017). As a result, the flavonoids have fascinated much attention of the scientists because they showed hepatoprotective activities and the therapeutic properties such as antioxidant, anti‐inflammatory, anticarcinogenic, and antiaging (Chen et al., 2020; Jabeen et al., 2019; Maleki et al., 2019; Vukovic et al., 2018). However, the peel of citrus fruits contains the abundant proportion of flavonoid as compared to other parts of the fruit. Unfortunately, it is thrown out as waste.

Meanwhile, gallic acid (3,4,5‐trihydroxybenzoic acid) is a kind of polyphenol that there is in plants, foods, and beverages. Furthermore, not only is it utilized for the biomedical fields, but also its antioxidant activities and the influence of corrosion protection on the steel surfaces have been studied (Ferraris et al., 2020). Therefore, the extraction of these bioactive compounds from peels is essential.

So far, the different approaches such as ultrasonic (Yu et al., 2019), eutectic solvents (Ming‐Zhua et al., 2020), colorimetric (Huang et al., 2018), supercritical fluids (Song et al., 2019), and adsorption (Zhang et al., 2019) have been reported for their extraction. Among these methods, adsorption is a simple and cost‐effective technique in comparison with other procedures since they have the intricate operation and high costs of process (Liu et al., 2021; Yao et al., 2021).

Graphene is a layered carbon nanostructure which sp^2^‐bonded carbon atoms are hexagonally arranged in it. The excellent physical and chemical properties of graphene caused that a lot of reports were presented about its different applications. Additionally, graphene oxide (GO) is other shape of graphene which has attracted lots of attentions from researchers since it can be used as a precursor for the significant production of graphene‐based materials (Liu et al., 2019). Meanwhile, GO has been introduced as an ideal adsorbent in the adsorption process of heavy metals and organic compounds from aquatic solutions since there are many functional groups such as, epoxy, hydroxyl, and carboxyl groups on it. In other words, the sorption sites on the GO are vast that can result in increasing adsorption capacity. Then, a large number of studies dedicated to this topic (Liu et al., 2019; Mohseni et al., 2017).

According to the main importance of adsorption process for phenolic compounds, we decided to present an easy and operative technique for the separation of bioactive compounds from lemon peel by GO. In fact, this project includes two steps. The first step involves adsorption and extraction of rutin, naringin, and gallic acid from lemon peel by GO. The second step includes the study of adsorption process of rutin by GO since it was the uppermost rate of flavonoid in lemon peel. Thus, the purpose of the present work was to compare sorption capacity of GO for the adsorption of bioactive materials in particular rutin from real sample (extract of the lemon peel) and the simulated rutin. Besides, the extracted flavonoids as antioxidant can be applied in future uses.

## 2 EXPERIMENTAL

### 2.1 Materials and methods

Citrus *limetta* was collected from local gardens. Rutin with HPLC grade, methanol, nitric acid, and acetic acid was purchased from Sigma‐Aldrich Inc., and graphene oxide nanoplatelets (99%, Thickness 3.4–7 nm with 6–10 Layers) were used as received. Fourier transform infrared spectroscopy (FTIR) was recorded using KBr tablets on a Thermo Nicolet Nexus 870 FTIR spectrometer. Scanning electron microscope (*SEM*) was taken using an MIRA3\\TESCAN‐XMU model. The samples were studied by thermogravimetric analysis (TGA) (Netzsch TG 209 F1 Iris1) under nitrogen gas atmosphere (10℃/min). The concentration of flavonoids was measured by variable‐wavelength UV‐Vis spectrophotometry of Unico UV 2100 Model. High‐performance liquid chromatography (HPLC) was performed with an Agilent Technologies 1200 liquid chromatograph which equipped to a quaternary solvent‐delivery system (an auto sampler and a UV detector). An Agilent Zorbax SB‐C18 column (100 × 4.6 mm, 5 μm particle size) was used for all analyses. An ultrasonic bath with temperature control (Elmasonic, S60H) was used to disperse the GO in solution.

### 2.2 Preparation of lemon peels

Lemons with an average weight of 100.0 ± 2.0 g were used. They were washed with the distilled water and then were manually peeled. The obtained peels were dried in an oven at 40℃ for 48 hr. Thus, the dried peels were milled using a blender (Molinex) to produce the lemon peel powder (LPP). LPP was packed and kept in refrigerator at 4℃ for use.

### 2.3 Preparation of the extract of lemon peel

5 g of LPP was blended with 30 ml of the ethanol–water mixture (70:30, v/v) as solvent. Then, the mixture was placed in the ultrasonic bath for 30 min at room temperature (25℃). The suspension was filtered with filter paper to remove the large particles, and the remaining powder was extracted for other two times using 25 ml of the ethanol–water mixture (70:30, v/v). After filtration, the filtrate was diluted to 100 ml in a volumetric flask (100 ml) by the ethanol–water mixture (70:30, v/v) and the color of the lemon peel extract was light yellow and the extract was stored at 4℃ for test.

### 2.4 Chromatographic conditions for the analysis of bioactive compounds

Chromatographic analyses were performed under gradient conditions. The components of mobile phase were methanol (A) and 0.1% acetic acid (B). Separation was performed using a gradient program: 0–8 min: 0 min, 15% A and 85% B; 4 min, 30% A and 70% B; 8 min, 45% A, 55% B; 12 min, 65% A, 35% B; 16 min, 45% A, 55% B; 20 min, 30% A, 70% B; 24 min, 15% A and 85% B. In addition, the time of re‐equilibration between runs was 10 min. The analysis was achieved at 30℃ (column oven temperature), with a 0.6 ml/min flow rate, and the injection volume was 5 μl. Chromatography peaks were characterized by comparing their retention time with that of the standard samples. Furthermore, diode‐array detection (DAD) in HPLC was used to make a spectral scan of the peaks (254–360 nm). All chromatography operations were carried out at ambient temperature and in triplicate. The linearity of the method was established using five standard solution levels for rutin (concentration range of 10–50 mg/L) evaluated in triplicate on three separate experiments. Standard calibration curve of rutin was calculated with linear equation of *Y* = 1.7343*X* + 0.4292 and correlation coefficient (*R* ^2^ = .9999) where *X* is the content of the rutin and *Y* is the peak height.

### 2.5 The effect of pH on the adsorption of the flavonoids

To study the effects of pH on the sorption of flavonoids, 50 mg of GO was dispersed into 15 ml of extract. The initial pH values were adjusted from 1.0 to 9.0 using nitric acid and NaOH at 25 ± 1°C. Then, the mixture was stirred for 5 min. As a result, their adsorption percentage was determined by UV‐Vis spectroscopy. According to Equation (1), adsorption percentage can be calculated as follows:

(1) $$\%A=\frac{A_{0}‐A_{e}}{A_{0}}\times 100$$

where A_0_ and A_e_ are the initial and final absorptions of these compounds in the solution, respectively. The results are based on at least three replicate experiments for each pH value.

### 2.6 Desorption of GO‐flavonoids

**FIGURE 1:**
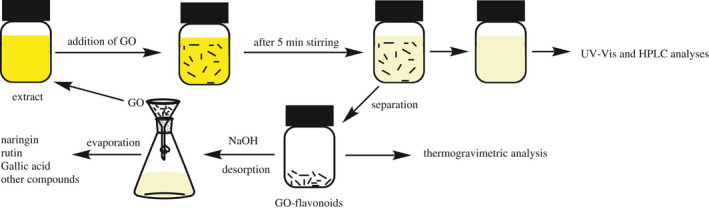
The procedure of the adsorption process of the different compounds from the extract of lemon peel by graphene oxide (GO) and the trend of getting UV‐Vis, High‐performance liquid chromatography (HPLC), and thermogravimetric analysis (TGA) analyses

The adsorption was carried out in 15 ml of extract with 50 mg of GO at 298 K, and then, the mixture was stirred for 5 min (the dosage and contact time values were optimized). After filtration, the GO‐flavonoids were immersed in 15 ml of ethanol–water (70:30, v/v) solution at pH = 9. Then, GO was separated from the solution and washed with water. Finally, the released flavonoid values in the solution were determined by HPLC. Figure 1 shows the procedure for the separation process of bioactive compounds, in particular flavonoids, by GO.

### 2.7 Batch sorption experiments of rutin

To study the effects of pH on the sorption of rutin, 50 mg of GO was dispersed into 15 ml solutions containing 100 mg/L of rutin with the various pH values (3–9) which these values were obtained by nitric acid and NaOH at 25 ± 1℃. The proportion of the absorbed rutin was calculated as the difference between the initial and final concentrations when the equilibrium was reached. The tests were done three times for each pH value. To evaluate the sorption capacity, 50 mg of GO was mixed with 15 ml of rutin solution (concentration range 10–100 mg/L). After 150 min, the rutin concentration in the aqueous solutions was determined by UV‐Vis spectroscopy. The removal (%) and sorption capacity q_e_ (mg/g) were acquired as follows in Equations (2) and (3):

(2) $$\text{adsorption}\;\%=\frac{C_{0}‐C_{e}}{C_{0}}\times 100$$

(3) $$q_{e}=\frac{\left(C_{0}‐C_{e}\right)\times V}{m}$$

where C_0_ and C_e_ are the initial and final concentrations (mg/L) of rutin in the aqueous solution, respectively, V (L) is the volume of rutin solution, and m (g) is the weight of sorbent. The kinetic experiments were carried out under normal atmospheric conditions at 25 ± 1℃. 50 mg of GO was contacted with 15 ml solution including 100 mg/L rutin concentration in glass vials, and then, it was stirred for the different times. Adsorbent and solution were separated at predetermined time intervals, filtered using a 0.45 µm membrane filter, and analyzed for residual rutin concentrations as described in above.

## 3 RESULTS AND DISCUSSION

### 3.1 Characterization of the GO

Figure 1 shows the procedure for the separation process of the flavonoids and gallic acid by GO. Different methods were utilized for the characterization of samples, such as FTIR, TGA, SEM, UV‐Vis, and HPLC which we explained in the report. FTIR spectrum of GO is shown in Figure S1. As can be observed, the peaks at 3,427, 1,732, 1,620, and 1,000–13,00 cm^−1^ can be related to OH, C = O, C = C, and C‐O stretching modes, respectively, that certainly indicated carboxylic and hydroxyl groups on the graphene.

**FIGURE 2:**
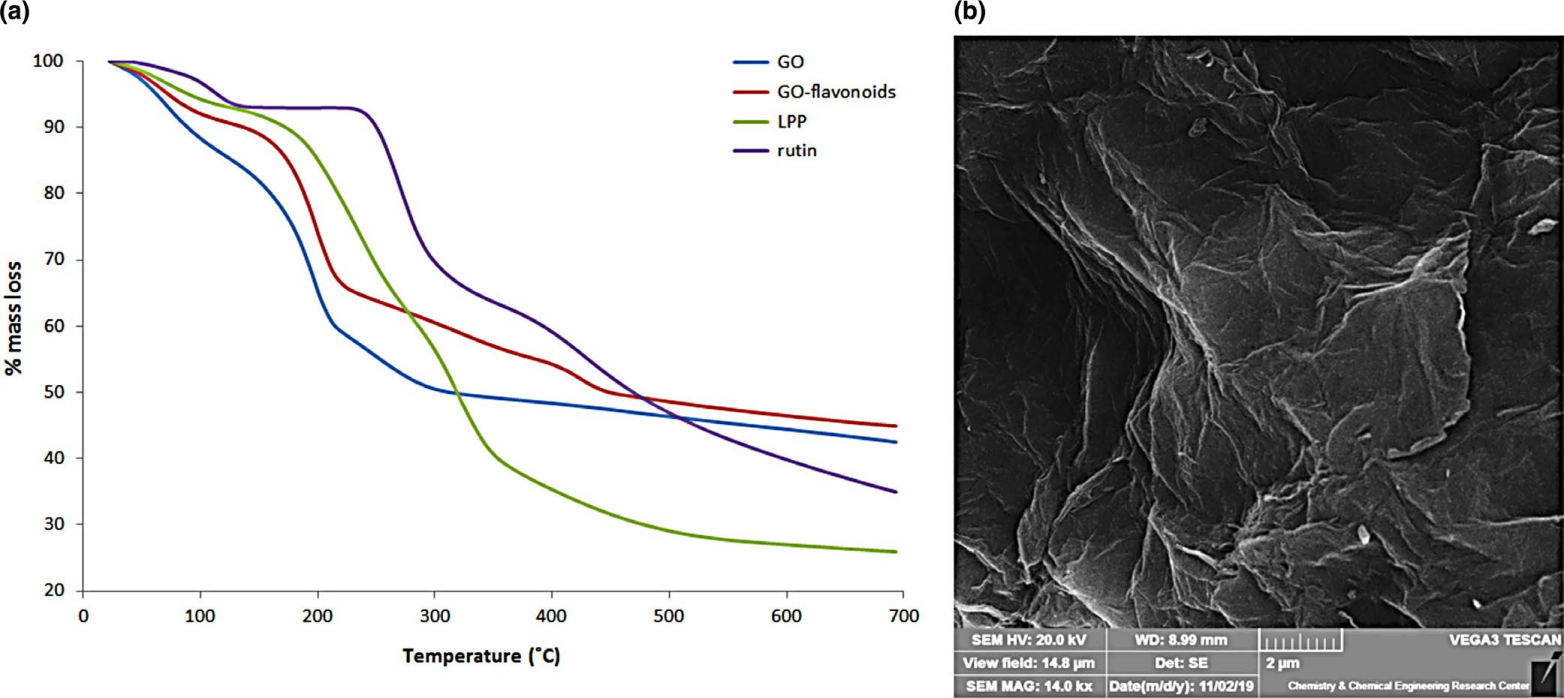
(a) Thermogravimetric analysis (TGA) curves of samples in the N_2_ (10 ˚C /min). (b) SEM image of graphene oxide (GO)

Thermogravimetric analysis is a thermal method in which the required temperatures for decomposition of functional groups attached to graphene are the more different than graphene. Thus, their weight loss in the various temperatures can be applied to evaluate the proportion of functional groups attached to graphene. Hence, it provides the useful information about the adsorbed flavonoids by GO. As it is observable in Figure 2a, each curve illustrates one decomposition below 130℃ which may be related to the evaporation of adsorbed water on the GO. TGA of GO indicates a decomposition between 140 and 330℃ with mass loss of 33.52% that can be probably assigned to the different oxygen‐containing functional groups on the GO. TGA curve of GO‐flavonoids exhibited two decompositions at around 130–250℃ with mass loss of 27.23% and 260–700℃ with mass loss of 19.36% that can be assigned to the residual oxygen‐containing groups and the different attached flavonoids, in particular rutin, respectively, on the GO as compared to thermograms of them. Moreover, our results revealed that the value about 0.76 mg of flavonoids was on the GO‐flavonoids (3.92 mg of GO‐flavonoids was used for test).

FESEM image of GO in Figure 2b presents graphene sheets which remarkably indicate the sheet‐like arrangement of graphene with huge wideness and plane surface. This showed that the treated sample had morphology of a graphene structure.

### 3.2 pH effect

**FIGURE 3:**
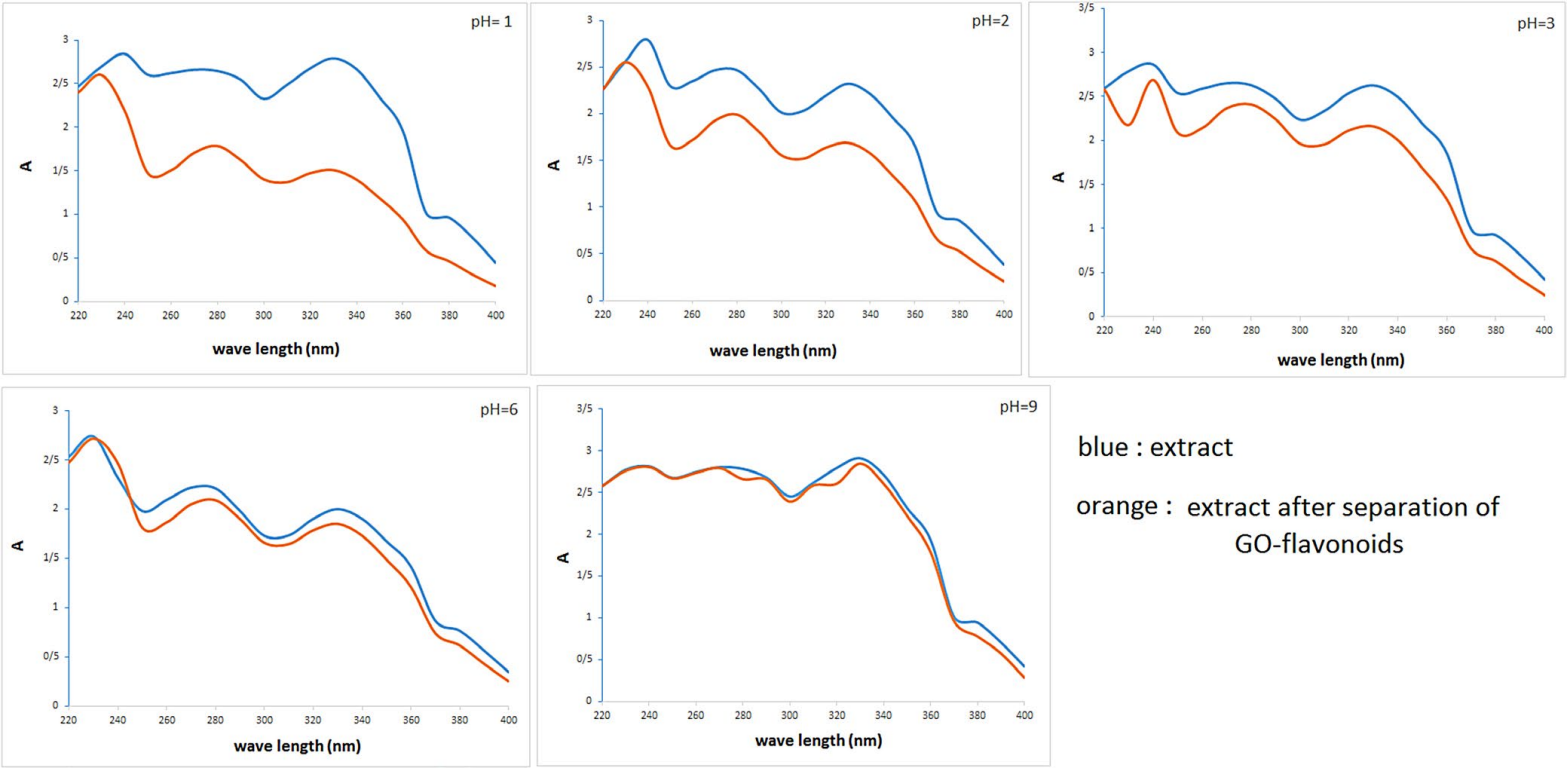
UV–Vis spectra of the extract and the separated extract of GO‐flavonoids in the different pH values

Figure 3 shows the absorption spectra (UV‐Vis) of extract (blue curve) and extract after the separation of GO‐flavonoids (orange curve) in the various pH values. The wavelength maximum (λ_max_) of the considered flavonoids was detected about 240–365 nm that they corresponded to literature (González‐Molina et al., 2010). According to Figure 3, diminishing pH values results in the absorption decrease of extract after the separation of GO‐flavonoids in comparison with extract. It means that the difference between two curves increased at lower pH. In other words, the adsorption maximum of bioactive compounds by GO was noticeable at pH = 1. In addition, adsorption percentage of the flavonoids was obtained in a range from 2.4 ± 0.8 to 46 ± 2.1% when pH changed from 9 to 1. The pH values play the important role in the process of adsorption as the considered flavonoids had a large number of hydroxyl groups. The surface functional groups of the flavonoids in high pH values are either neutral or negatively charged since they dissociate to their anions. Then, decreasing adsorption capacities because of the electrostatic repulsion between the identical charges is recognizable. Meanwhile, being more soluble anions at pH = 9 causes the strong interaction between adsorbate and water which avoid adsorbing flavonoids by GO. So, the adsorption of flavonoids dropped in the higher pH.

### 3.3 Desorption performance of GO

**FIGURE 4:**
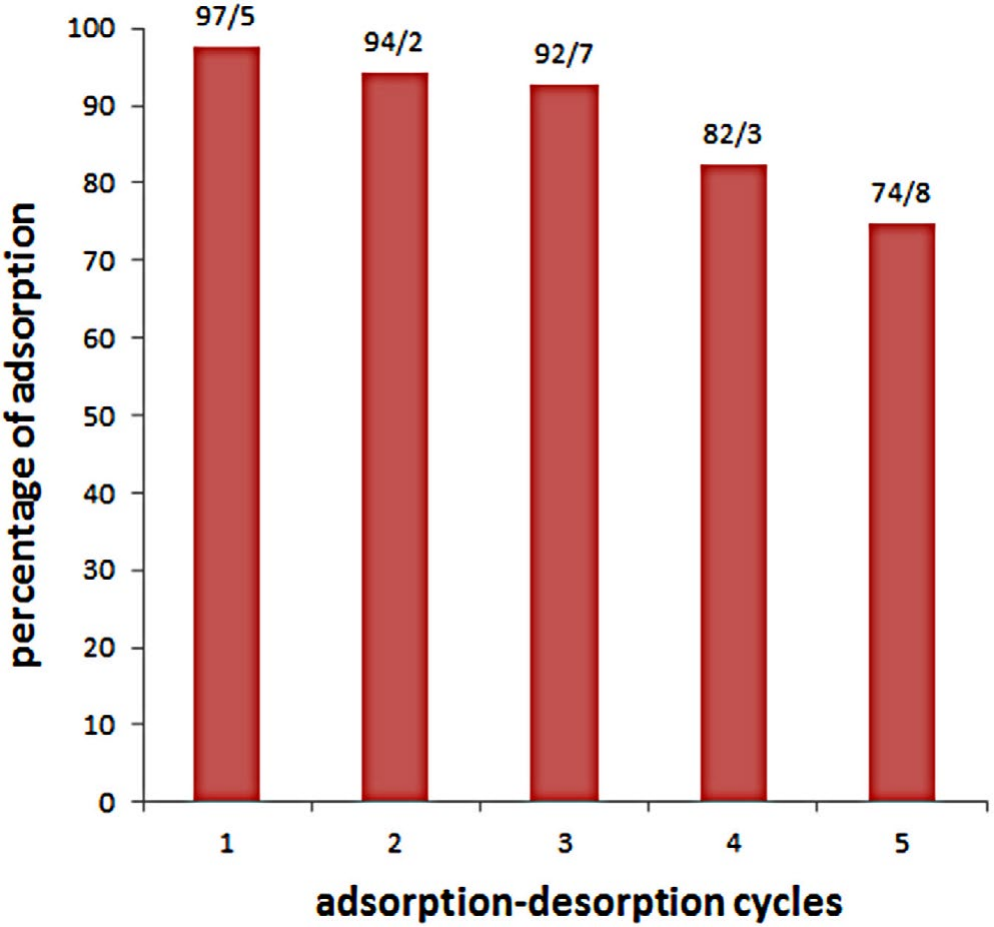
The adsorption–desorption cycles of the different compounds by graphene oxide (GO) from extract

The adsorption–desorption processes are vital factors for an adsorbent since they can decline the entire price for efficiency of the adsorbents. On the basis of our previous article (Gholizadeh et al., 2020), the best results of desorption were obtained when pH reached 9. Therefore, we carried out the desorption experiments at pH = 9. Figure 4 shows the results of the adsorption–desorption cycles of GO. As can be seen in Figure 4, GO can be reused for 4 cycle since the adsorption capacity of bioactive compounds decreased about 18 ± 2.9% up to cycle 4. In addition, it can be perceived that GO with the remarkable steadiness till the third cycle can be utilized as an outstanding recyclable adsorbent for these substances.

### 3.4 The adsorption mechanism of bioactive compounds by GO

**FIGURE 5:**
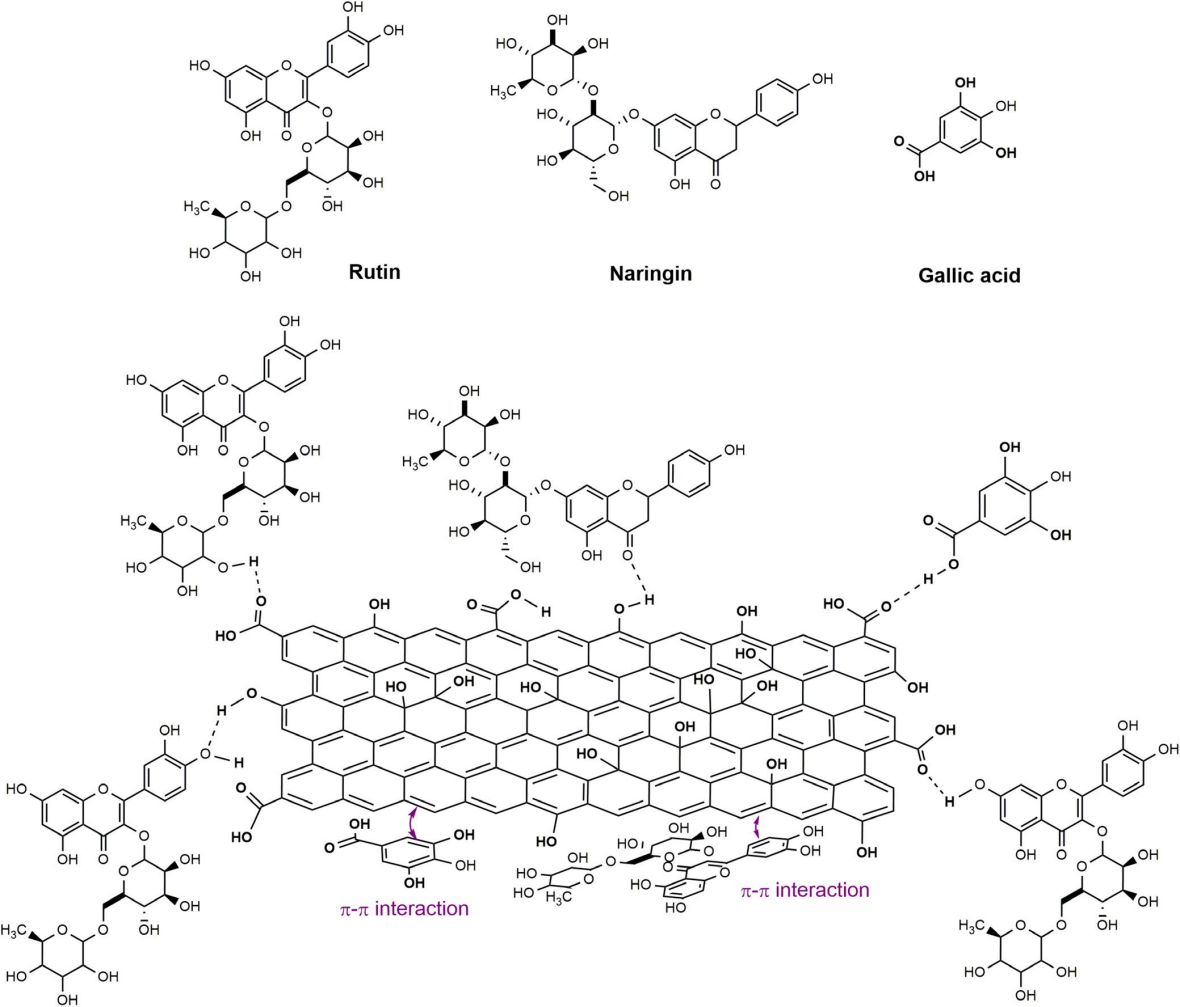
The proposed mechanisms for adsorption of the different compounds on the graphene oxide (GO)

The structures of bioactive compounds (naringin, rutin, and gallic acid) of extract along with the offered mechanism for the adsorption of them by GO are displayed in Figure 5. As can be seen, there are a lot of polar functional groups such as hydroxyl and carbonyl in rutin, naringin, gallic acid, and GO. Hence, they tend to form hydrogen bonds together. In fact, one of the main mechanisms for the adsorption of bioactive compounds from extract is hydrogen bond. On the basis, adsorption process of bioactive compounds of extract by GO can be related to the hydrogen bond interactions between the oxygen of a bioactive compound and the hydrogen of an alcohol or carboxylic group of GO and vice versa. Additionally, the π‐π interactions between the double bonds of graphene and aromatic ring of the bioactive compounds can be other factor for the adsorption process. These interactions are shown in Figure 5. Then, these proposed mechanisms can culminate in the adsorption of rutin, naringin, and gallic acid by GO.

### 3.5 HPLC analysis of bioactive compounds from lemon peel

**FIGURE 6:**
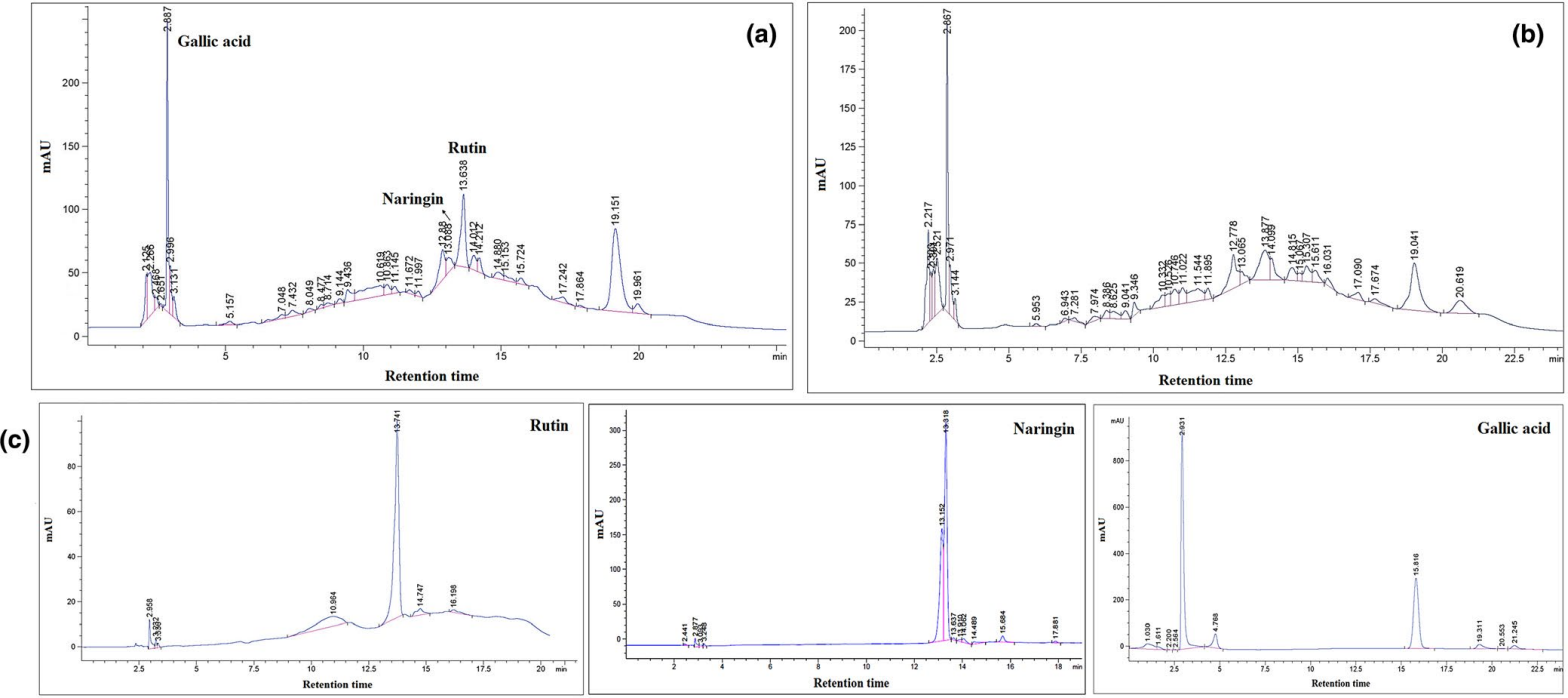
Chromatograms were as follows: (a) the crude extract; (b) the extract after separation of GO‐flavonoids from it; and (c) the standard references of samples

Graphene oxide performance for the separation of flavonoids was considered based on the rutin and naringin. The obtained HPLC data were shown in Figure 6. As Figure 6a presented, the highest flavonoid proportion of extract was rutin and that all of the bioactive compounds were well separated. Figure 6b,c illustrates the HPLC results of the extract after the filtration of GO‐flavonoids from solution and the standard samples, respectively. In relation to Figure 6c, the elution order was gallic acid, naringin, and rutin with the retention times at 2.8, 13.24, and 13.74 min, respectively. As can be witnessed in Figure 6b, the findings of HPLC analysis exhibited that the GO culminate in sorption of rutin from extract about 22.16 ppm since it was appraised about 32.97 ppm in the extract on the basis of standard calibration curve. Therefore, the percentage of the rutin extraction was obtained about 66.7%. On the other hand, the peak height of naringin (13.08 min) and gallic acid (2.8 min) of extract was 12.01 (mAU) and 229.4 (mAU), respectively, while these values for the separated extract from GO‐flavonoids were calculated 8.02 (mAU) for naringin and 186.2 (mAU) for gallic acid. Hence, with the comparison of HPLC data, we can draw the conclusion that the GO results in sorption of naringin and gallic acid from extract about 34% and 19%, respectively. Although the naringin, rutin, and gallic acid were characterized, an unknown compound with the retention time at 19 min was meaningfully adsorbed by GO about 46.6% which it may be a flavonoid. Totally, GO can significantly cause the extraction of the different flavonoids, which this is a great result.

Since the most significant rate of flavonoid in lemon peel was rutin, we decided to investigate the adsorption process for the simulated samples of it. In other words, our purpose is the study of the sorption mechanism of rutin on the GO and the comparison with the extract sample.

### 3.6 Adsorption studies of rutin

#### 3.6.1 pH effect

**FIGURE 7:**
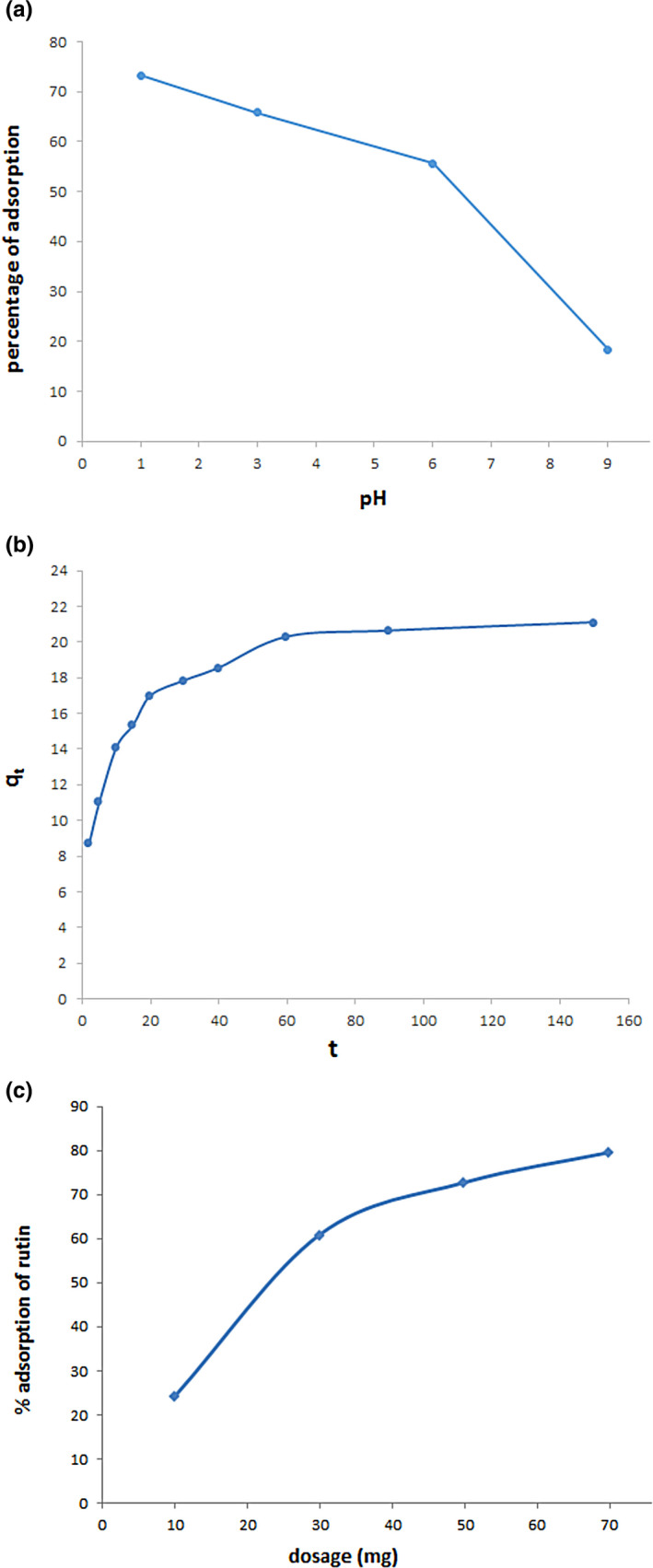
(a) Effect of initial pH on rutin sorption onto graphene oxide (GO). (b) Effect of contact time on the adsorption of rutin by GO (experimental conditions: pH = 1; adsorbent mass, 50 mg/15 ml; rutin concentration, 100 mg/L). (c) Effect of adsorbent dosage on the adsorption of rutin from solution (experimental conditions: pH = 1; contact time, 150 min; rutin concentration, 100 mg/L, volume, 15 ml)

We studied pH effect on the adsorption of rutin by GO as was exhibited in Figure 7a. As can be understood in Figure 7a, we witnessed a 54.8% growth of the adsorption from pH = 9 to pH = 1 and got to nearly 73.2 ± 2.3%. Since there is an inversion relation between the adsorption and solubility in phenolic organic species (Gholitabar et al., 2017), we can draw the conclusion that falling pH values decreased the rutin solubility and thus culminate in the increase of the adsorption percentage. In comparison, these values reduced with rising the pH values so that the quantity of the adsorption reached 18.4 ± 0.9% at pH = 9. Additionally, the electrostatic repulsion between the negative‐charged formed groups in the surface of GO and rutin at pH = 9 significantly declined the adsorption percentage. Furthermore, the existence of more soluble rutin at pH = 9 causes the strong interaction between adsorbate and water which it prevents adsorbing rutin by GO.

#### 3.6.2 Sorption kinetic

The influence of contact time on the adsorption of the rutin by GO was indicated in Figure 7b. At up to 20 min of initial contact time, the rutin adsorption rate was relatively rapid and then reached equilibrium at nearly 90 min. The final value of the adsorption capacity was found to be 20.62 mg/g at equilibrium time. As can be seen in Figure 7b, the rutin sorption considerably augmented with increasing the initial contact time (up to 20 min) which shows the high interaction of GO with rutin. The rapid sorption at the initial contact time can be attributed to the presence of a great many of vacant adsorption sites such as carboxyl and hydroxyl on the adsorbent surface, in which rutin molecules can interact easily with these sites. After 60 time, the remaining available sites were difficult for the occupancy of rutin molecules which it can be related to the repulsion between the adsorbed rutin molecules on the GO. Besides, rutin molecules can enter into the pores of the adsorbent and were adsorbed by the interior surface provided the adsorption of exterior surface reached saturation (90 min).

Table 1 shows kinetic models for the adsorption process of rutin by GO (Zhao et al., 2020). According to Table 1, the pseudo‐second‐order model will be able to describe the adsorption process of rutin on the GO since this model showed the higher correlation coefficient (0.9994) than pseudo‐first‐order model (0.9694). Moreover, the adsorbed values of rutin at equilibrium (q_e_) by GO were found 21.79 mg/g which significantly approved with the corresponded experimental data (q_e,ex_ = 21.081). In addition, the plots of adsorption kinetic models were presented in Supplementary Data.

**TABLE 1:** The calculated parameters of pseudo‐first‐order and pseudo‐second‐order models of rutin sorption onto graphene oxide (GO)

Kinetic models					
	The calculated parameters			Plot	q_e,ex_
Pseudo‐first‐order					21.08
$\log\;\left(q_{e}‐q_{t}\right)=\log\;\left(q_{e}\right)‐\frac{k_{1}}{2.303}t$	q_e_(mg/g)	k_1_ (min^−1^)	R^2^	log (*q_e_*‐*q_t_*) versus *t*	
	10.59	0.0375	0.9694		
Pseudo‐second‐order					
$\frac{t}{q_{t}}=\frac{1}{\left(k_{2}q_{e}^{2}\right)}+\frac{1}{q_{e}}t$	q_e_(mg/g)	k_2_ (g/mg min^−1^)	R^2^	*t*/*q_t_* versus *t*.	
	21.79	0.0086	0.9994		

Note

Temperature, 298 K; initial rutin concentration, 100 mg/L; mass of GO, 50 mg; volume of solution, 15 ml; and pH of the sample solution, 1.0.

#### 3.6.3 The dosage effect of adsorbent

Figure 7c shows the dosage effect of GO on the adsorption percentage of rutin. The empirical findings showed a gradual rise in the adsorption of rutin with rising GO dosage from 0.01 to 0.07 g. This increase of the dosage noticeably enhanced the adsorption percentage of rutin from 24.2 ± 2.4% to 79.5 ± 3.5%. These results can be related to the fact that the dosage growth of GO makes more available adsorption sites for the rutin.

#### 3.6.4 Adsorption isotherms

We use adsorption isotherms, which are mathematical equations, whenever the relationship of the adsorbate concentrations in the solid and liquid media is important for us since they describe how the adsorbate species adsorbed to the adsorbent surface (Foo et al., 2010). As a matter of fact, adsorption isotherms show that when an equilibrium state is established in system, how a substance from the aqueous media transfers to a solid phase. Three adsorption isotherm models, Langmuir, Freundlich, and Dubinin–Radushkevich, have been utilized to describe the mechanism of adsorption.

Langmuir isotherm assumes monolayer adsorption of the molecules on the adsorbent surface with the same activation energy of adsorption. However, it is used for the homogeneous surface (Massoud et al., 2020). Besides, multilayer adsorption and the heterogeneous surfaces with nonuniform distribution of adsorption heat are the features of Freundlich isotherms (Zhang et al. 2019). These models along with their calculated parameters, which describe the surface properties and affinity of the adsorbent, are indicated in Table 2. All plots of adsorption isotherms are presented in Supplementary Data.

**TABLE 2:** The parameters of the different isotherm models for rutin sorption from solution by graphene oxide (GO)

Isotherm models	The calculated parameters				Plot
Langmuir	q_m_(mg/g)	b (L/mg)	*R* ^2^		C_e_/q_e_ versus C_e_
$\frac{C_{e}}{q_{e}}=\frac{1}{{\text{bq}}_{m}}+\frac{C_{e}}{q_{m}}$	22.32	0.739	0.9934		
Freundlich	K_f_ (mg/g) (mg/L)*^n^*	*n*	*R* ^2^		ln q_e_ versus ln C_e_
$\ln\;q_{e}=\ln\;K_{f}+\frac{1}{n}\;\ln\;C_{e}$	8.728	3.302	0.9659		
Dubinin–Radushkevich	q_m_ (mg/g)	K_DR_	*R* ^2^	E (kJ/mol)	ln q_e_ versus *ε* ^2^
$\ln\;\left(q_{e}\right)=\ln\;q_{m}‐K_{\text{DR}}\varepsilon^{2}$	16.87	3.14 × 10^–8^	0.8910	3.989	

As Table 2 shows, Langmuir isotherm with high correlation coefficient (0.9934) better than Freundlich model (0.9659) described the adsorption process of rutin by GO. In other words, rutin adsorption was the monolayer and equal activation energy was considered for sorption of rutin molecules on the GO.

On the other hand, Dubinin–Radushkevich (D‐R) isotherm describes a Gaussian energy distribution onto a heterogeneous surface which it is utilized to explain the adsorption mechanism (Brohi et al., 2020). It can be expressed as follows in Equations (4) and (5):

(4) $$\ln\left(q_{e}\right)=\ln\;q_{m}‐K_{\text{DR}}\varepsilon^{2}$$

(5) $$\varepsilon =RT\ln\left(1+\frac{1}{C_{e}}\right)$$

where q_e_ (mg/g) is the volume of rutin adsorbed, q_m_ (mg/g) is the maximum adsorption capacity of rutin, *K*_DR_ (mol^2^/kJ^2^) is the D‐R isotherm constant, *ε* is Polanyi potential, *R* is the gas constant (0.008314 kJ/mol K), and *T* (K) is absolute temperature in Kelvin. Meanwhile, the constant K_D_ presents the mean free energy, *E* (kJ/mol), of sorption per molecule of sorbate when it is transferred to the surface of the solid from infinity in the solution which can be estimated using the following Equation (6):

(6) $$E=\frac{1}{\sqrt{2K_{D}}}$$

In fact, the *E* value describes whether the adsorption on the GO has happened as a physical or chemical process which it is important. On the basis of some papers (Foo et al., 2010; Kausar et al., 2013), the adsorption process can be considered as the physical adsorption if the calculated value of E is below 8 kJ/mol but the chemical adsorption occurs in the range of 8–16 kJ/mol. As can be seen in Table 2, the calculated proportion of *E* was 3.9 kJ/mol. Then, it can be decided that physical adsorption may play a substantial role in the adsorption process of rutin by GO.

### 3.7 GO efficiency for the rutin adsorption from extract and the simulated sample

The acquired findings showed that GO efficiency for rutin sorption from the simulated solution (standard solution: 100 ppm) and the lemon peel extract (32.97 ppm) was about 72 ± 1.6% and 67 ± 2.5%, respectively, since these experiments carried out in the same conditions (solution volume: 15 ml, dosage: 50 mg). With regard to the presence of the various compounds in lemon peel such as naringin, rutin, gallic acid, and even other materials, which did not determine, GO efficiency for rutin sorption can be suitable and worthwhile in the range of the considered concentrations since in addition to rutin, naringin, and gallic acid were also adsorbed and separated by GO.

## 4 CONCLUSIONS

In this project, we described a method for separation of flavonoids of lemon peel by GO. The desorption percentage of flavonoids was 74.8% after five cycle. Furthermore, the rutin quantity which was adsorbed from extract by GO was almost similar to that of the simulated rutin in terms of the adsorption percentage. These results may be able to be valuable in future applications. Moreover, we analyzed the process of rutin adsorption by GO. Langmuir isotherm meaningfully interpreted the adsorption process of rutin. The pseudo‐second‐order kinetic showed the admissible description from the process.

## CONFLICTS OF INTEREST

All authors declare that there is no conflict of interest.

## AUTHOR CONTRIBUTIONS

**Valeh Sharif Nasirian :** Investigation (lead). **Seyed‐Ahmad Shahidi:** Supervision (equal). **Hasan Tahermansouri:** Project administration (equal); Supervision (lead). **Fereshteh Chekin:** Validation (equal).

## ETHICAL APPROVAL

This article does not contain any studies with human participants or animals performed by any of the authors.

## Supporting information

Supplementary Material

## Data Availability

Data available on request due to privacy/ethical restrictions.

## References

[fsn32363-bib-0001] Adibelli, Z. , Dilek, M. , & Akpolat, T. (2009). Lemon juice as an alternative therapy in hypertension in Turkey. International Journal of Cardiology, 135, e58–e59. 10.1016/j.ijcard.2008.03.085 18597876

[fsn32363-bib-0002] Benavente‐Garcia, O. , & Castillo, J. (2008). Update on uses and properties of Citrus flavonoids: New findings in anticancer, cardiovascular, and anti‐inflammatory activity. Journal of Agricultural and Food Chemistry, 56, 6185–6205. 10.1021/jf8006568 18593176

[fsn32363-bib-0003] Brohi, R. O. Z. , Khuhawar, M. Y. , & Mahar, R. B. (2020). Graphene oxide functionalized with a Schiff Base for the removal of Pb(II) ions from contaminated water: Experimental and modeling approach. Journal of Chemical Technology & Biotechnology, 95, 1694–1704. 10.1002/jctb.6362

[fsn32363-bib-0004] Chen, H. , Wang, J. , Liu, X. , Zhou, A. , Xiao, J. , Huang, K. , Chen, H. , & Cao, Y. (2020). Optimization in continuous phase‐transition extraction of crude flavonoids from finger citron fruit and evaluation on their antiaging activities. Food Science & Nutrition, 8, 1636–1648. 10.1002/fsn3.1450 32180971 PMC7063346

[fsn32363-bib-0005] El‐desoky, A. H. , Abdel‐Rahman, R. F. , Ahmed, O. K. , El‐Beltagi, H. S. , & Hattori, M. (2018). Anti‐inflammatory and antioxidant activities of naringin isolated from Carissa carandas L.: In vitro and in vivo evidence. Phytomedicine, 42, 126–134. 10.1016/j.phymed.2018.03.051 29655678

[fsn32363-bib-0006] Ferraris, S. , Cazzola, M. , Ubertalli, G. , Prenesti, E. , & Spriano, S. (2020). Grafting of gallic acid to metallic surfaces. Applied Surface Science, 511, e145615. 10.1016/j.apsusc.2020.145615

[fsn32363-bib-0007] Foo, K. Y. , & Hameed, B. H. (2010). Insights into the modeling of adsorption isotherm systems. Chemical Engineering Journal, 156, 2–10. 10.1016/j.cej.2009.09.013

[fsn32363-bib-0008] Gholitabar, S. , & Tahermansouri, H. (2017). Kinetic and multi‐parameter isotherm studies of picric acid removal from aqueous solutions by carboxylated multi‐walled carbon nanotubes in the presence and absence of ultrasound. Carbon Letters, 22, 14–24. 10.5714/CL.2017.22.014

[fsn32363-bib-0009] Gholizadeh, H. , Ghorban‐HasanSaraei, A. , Tahermansouri, H. , & Shahidi, S. A. (2020). The mechanism studies of the adsorption‐desorption process of rutin from water/ethanol solution and the extract of bitter orange peel by the carboxylated multiwalled carbon nanotubes. Journal of the Chinese Chemical Society, 67, 546–557. 10.1002/jccs.201900303

[fsn32363-bib-0010] González‐Molina, E. , Domínguez‐Perles, R. , Moreno, Da , & García‐Viguera, C. (2010). Natural bioactive compounds of Citrus limon for food and health. Journal of Pharmaceutical and Biomedical Analysis, 51, 327–345. 10.1016/j.jpba.2009.07.027 19748198

[fsn32363-bib-0011] Gullón, B. , Lú‐Chau, T. A. , Moreira, M. T. , Lema, J. M. , & Eibes, G. (2017). Rutin: A review on extraction, identification and purification methods, biological activities and approaches to enhance its bioavailability. Trends in Food Science & Technology, 67, 220–235. 10.1016/j.tifs.2017.07.008

[fsn32363-bib-0012] Huang, R. , Wu, W. , Shen, S. , Fan, J. , Chang, Y. , Chen, S. , & Ye, X. (2018). Evaluation of colorimetric methods for quantification of citrus flavonoids to avoid misuse. Analytical Methods, 10, 2575–2587. 10.1039/C8AY00661J

[fsn32363-bib-0013] Jabeen, E. , Janjua, N. K. , Ahmed, S. , Ali, T. , Murtaza, I. , Ashraf, Z. , Masood, N. , & Kalsoom, S. (2019). Antioxidant activity and hepatotoxicity of flavonoids and their metal complexes through co‐administration of β‐cyclodextrin. ChemistrySelect, 4, 9420–9432. 10.1002/slct.201902124

[fsn32363-bib-0014] Kausar, A. , Bhatti, H. N. , & MacKinnon, G. (2013). Equilibrium, kinetic and thermodynamic studies on the removal of U (VI) by low cost agricultural waste. Colloids and Surfaces B: Biointerfaces, 111, 124–133. 10.1016/j.colsurfb.2013.05.028 23787279

[fsn32363-bib-0015] Liu, X. , Ma, R. , Wang, X. , Ma, Y. , Yang, Y. , Zhuang, L. , Zhang, S. , Jehan, R. , Chen, J. , & Wang, X. (2019). Graphene oxide‐based materials for efficient removal of heavy metal ions from aqueous solution: A review. Environmental Pollution, 252, 62–73. 10.1016/j.envpol.2019.05.050 31146239

[fsn32363-bib-0016] Liu, X. , Pang, H. , Liu, X. , Li, Q. , Zhang, N. , Mao, L. , Qiu, M. , Hu, B. , Yang, H. , & Wang, X. I. (2021). Orderly porous covalent organic frameworks‐based materials: superior adsorbents for pollutants removal from aqueous solutions. The Innovation, 2, e100076. 10.1016/j.xinn.2021.100076 PMC8454561 34557733

[fsn32363-bib-0017] Maleki, S. J. , Crespo, J. F. , & Cabanillas, B. (2019). Anti‐inflammatory effects of flavonoids. Food Chemistry, 299, e125124. 10.1016/j.foodchem.2019.125124 31288163

[fsn32363-bib-0018] Mallepu, R. , Potlapally, L. , & Gollapalli, V. L. (2018). Photo‐oxidation of some flavonoids with photochemically generated t‐BuO• radicals in a t‐BuOH water system using a kinetic approach. Journal of the Chinese Chemical Society, 65, 1266–1273. 10.1002/jccs.201700342

[fsn32363-bib-0019] Manjunatha, A. S. , Pavithra, N. S. , Marappa, S. , Prashanth, S. A. , Nagaraju, G. , & Nagaraju, P. (2018). Green synthesis of flower‐like BiVO4 nanoparticles by solution combustion method using lemon (citrus limon) juice as a fuel: Photocatalytic and electrochemical study. ChemistrySelect, 3, 13456–13463. 10.1002/slct.201801853

[fsn32363-bib-0020] Massoud, R. , Khosravi‐Darani, K. , Sharifan, A. , Asadi, G. H. , & Zoghi, A. (2020). Lead and cadmium biosorption from milk by Lactobacillus acidophilus ATCC 4356. Food Science & Nutrition, 8, 5284–5291. 10.1002/fsn3.1825 33133531 PMC7590288

[fsn32363-bib-0021] Ming‐Zhua, G. , Qi, C. , Li‐Tao, W. , Yao, M. , Lian, Y. , Yan‐Yan, L. , & Yu‐Jie, F. (2020). A green and integrated strategy for enhanced phenolic compounds extraction from mulberry (Morus alba L.) leaves by deep eutectic solvent. Microchemical Journal, 154, e104598. 10.1016/j.microc.2020.104598

[fsn32363-bib-0022] Mohseni Kafshgari, M. , & Tahermansouri, H. (2017). Development of a graphene oxide/chitosan nanocomposite for the removal of picric acid from aqueous solutions: Study of sorption parameters. Colloids and Surfaces B: Biointerfaces, 160, 671–681. 10.1016/j.colsurfb.2017.10.019 29031227

[fsn32363-bib-0023] Musawwer Khan, M. , Khan, S. , Saigal, B. , & Sahoo, S. C. (2018). Efficient and eco‐friendly one‐pot synthesis of functionalized furan‐2‐one, pyrrol‐2‐one, and tetrahydropyridine using lemon juice as a biodegradable catalyst. ChemistrySelect, 3, 1371–1380. 10.1002/slct.201702933

[fsn32363-bib-0024] Panche, A. N. , Diwan, A. D. , & Chandra, S. R. (2016). Flavonoids: An overview. Journal of. Nutritional Science, 5, e47. 10.1017/jns.2016.41 28620474 PMC5465813

[fsn32363-bib-0025] Silva Antonio, A. , Wiedemann, L. S. M. , & Veiga‐Junior, V. F. (2020). Natural products' role against COVID‐19. RSC Advances, 10, 23379–23393. 10.1039/D0RA03774E PMC9122563 35693131

[fsn32363-bib-0026] Song, L. , Liu, P. , Yan, Y. , Huang, Y. , Bai, B. , Hou, X. , & Zhang, L. (2019). Supercritical CO_2_ fluid extraction of flavonoid compounds from Xinjiang jujube (Ziziphus jujuba Mill.) leaves and associated biological activities and flavonoid compositions. Industrial Crops and Products, 139, e111508. 10.1016/j.indcrop.2019.111508

[fsn32363-bib-0027] Stefova, M. , Stafilov, T. , & Kulevanova, S. (2003). HPLC analysis of flavonoids. In Jack Cazes , ed. Encyclopedia of chromatography (pp. 183–195): Marcel Dekker.

[fsn32363-bib-0028] Talib, W. H. (2017). Consumption of garlic and lemon aqueous extracts combination reduces tumor burden by angiogenesis inhibition, apoptosis induction, and immune system modulation. Nutrition, 43–44, 89–97. 10.1016/j.nut.2017.06.015 28935151

[fsn32363-bib-0029] Vukovic, N. L. , Obradovic, A. D. , Vukic, M. D. , Jovanovic, D. , & Djurdjevic, P. M. (2018). Cytotoxic, proapoptotic and antioxidative potential of flavonoids isolated from propolis against colon(HCT‐116) and breast (MDA‐MB‐231) cancer cell lines. Food Research International, 106, 71–80. 10.1016/j.foodres.2017.12.056 29579978

[fsn32363-bib-0030] Wang, Y. C. , Chuang, Y. C. , & Hsu, H. W. (2008). The flavonoid, carotenoid and pectin content in peels of citrus cultivated in Taiwan. Food Chemistry, 106, 277–284. 10.1016/j.foodchem.2007.05.086

[fsn32363-bib-0031] Yao, L. , Yang, H. , Chen, Z. , Qiu, M. , Hu, B. , & Wang, X. (2021). Bismuth oxychloride‐based materials for the removal of organic pollutants in wastewater. Chemosphere, 273, e128576. 10.1016/j.chemosphere.2020.128576 34756376

[fsn32363-bib-0032] Yu, M. , Wang, B. , Qi, Z. , Xin, G. , & Li, W. (2019). Response surface method was used to optimize the ultrasonic assisted extraction of flavonoids from Crinum asiaticum. Saudi Journal of Biological Sciences, 26, 2079–2084. 10.1016/j.sjbs.2019.09.018 31889798 PMC6923458

[fsn32363-bib-0033] Zhang, L. , Zheng, D. , & Zhang, Q.‐F. (2019). Purification of total flavonoids from *Rhizoma Smilacis Glabrae* through cyclodextrin‐assisted extraction and resin adsorption. Food Science & Nutrition, 7, 449–456. 10.1002/fsn3.809 30847122 PMC6392876

[fsn32363-bib-0034] Zhao, L. , Li, X. , Yang, Q. , Zhuang, D. , Pan, X. , & Li, L. (2020). Adsorption kinetics and mechanism of di‐n‐butyl phthalate by Leuconostoc mesenteroides. Food Science & Nutrition, 8, 6153–6163. 10.1002/fsn3.1908 33282266 PMC7684587

