# Supplementary material for: Application of graphene oxide in the adsorption and extraction of bioactive compounds from lemon peel
Source: Food Sci Nutr. 2021 Jun 2;9(7):3852–62. doi: 10.1002/fsn3.2363 (PMC8269583; doi:10.1002/fsn3.2363)

**Application of graphene oxide in the adsorption and extraction of bioactive compounds from lemon peel**

Valeh Sharif Nasirian, Seyed-Ahmad Shahidi, Hasan Tahermansouri, Fereshteh Chekin

**Contents:**

Figure S1.

The plots of kinetic models of GO for adsorption of rutin

The plots of isotherm models of GO for adsorption of rutin


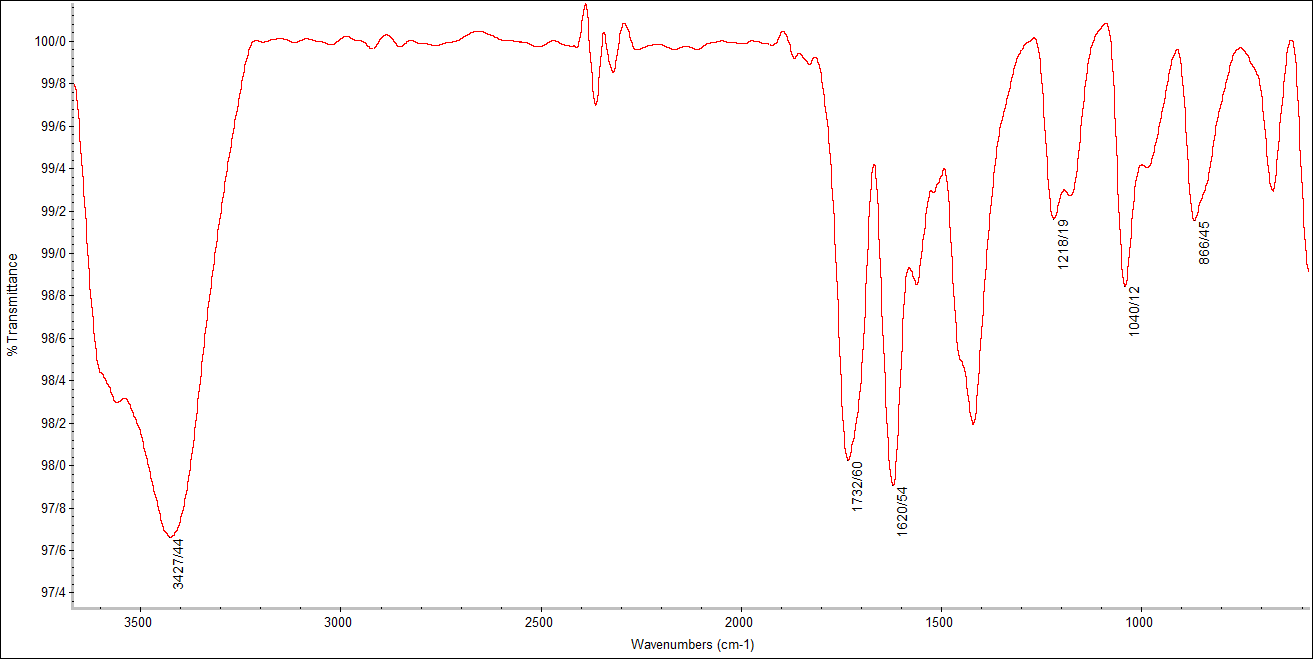


Figure S1. FT-IR spectrum (after baseline correction) of GO.


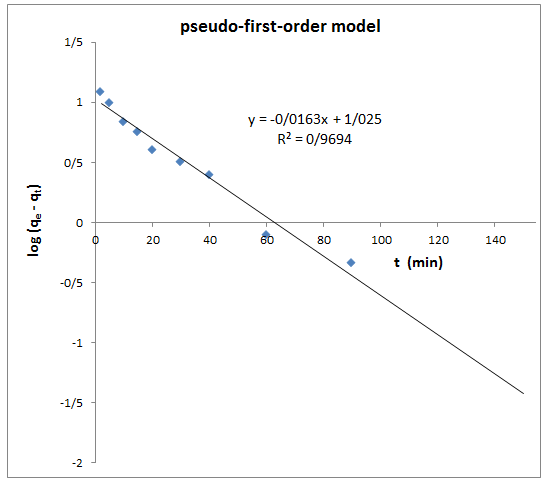


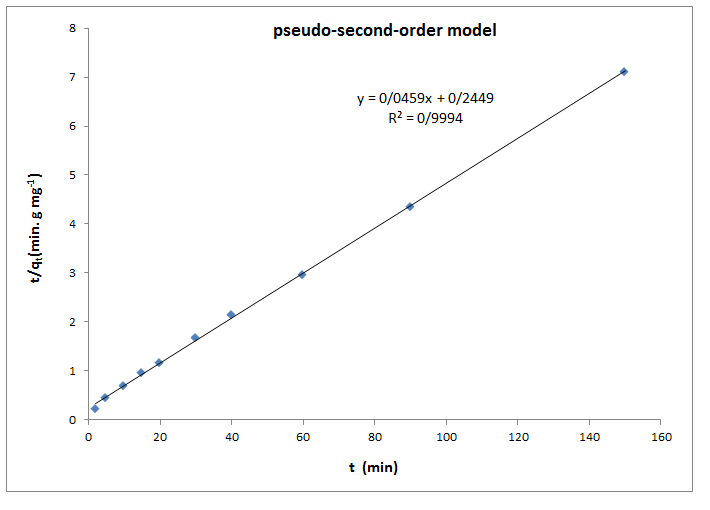


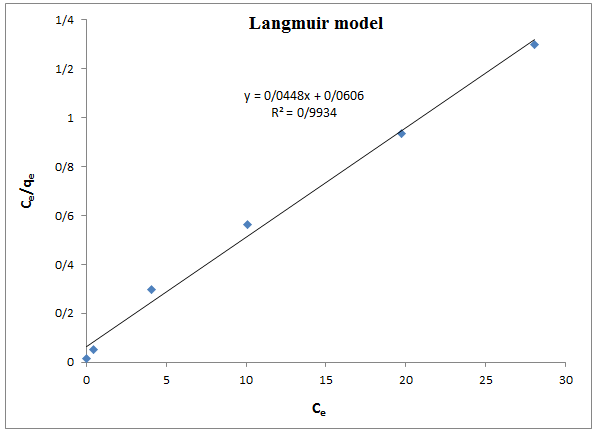


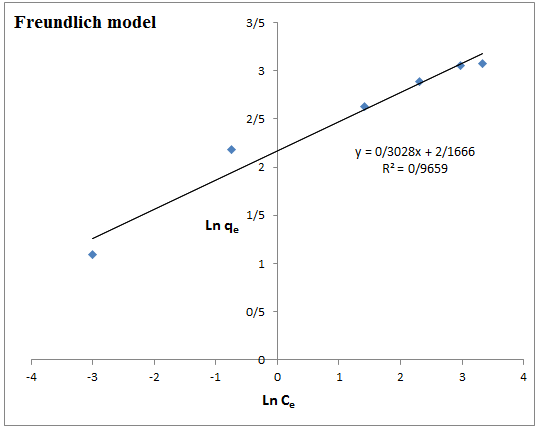


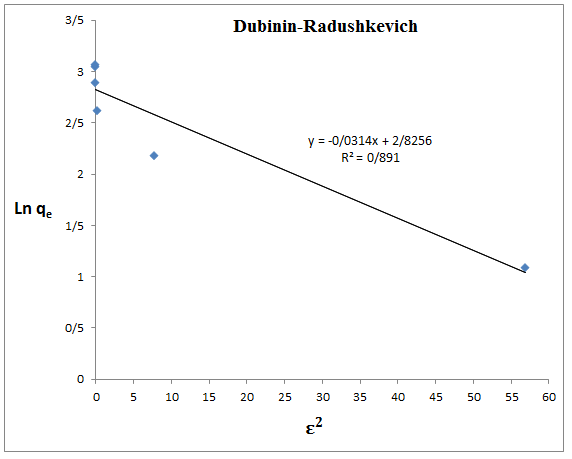

Supplement: Supplementary file 1 — Supplementary Material [file FSN3-9-3852-s001.docx]
